# Breast Tuberculosis: A Case Report

**DOI:** 10.7759/cureus.34175

**Published:** 2023-01-25

**Authors:** Margarida Pimentel Nunes, Inês Branco Carvalho, Isabel Araújo, Raquel Almeida, José Araújo

**Affiliations:** 1 Internal Medicine Department, Hospital Beatriz Ângelo, Loures, PRT

**Keywords:** breast abscess, scrofula, mycobacterium tuberculosis., mastitis, breast cancer differential diagnosis, breast tuberculosis

## Abstract

Breast tuberculosis (BTB) is a rare manifestation of tuberculosis (TB), and it is more common in countries with a high incidence of TB. We describe a case of a 36-year-old Angolan woman, who had a history of breast reduction surgery, presenting with right breast enlargement, pain, purulent discharge through multiple skin openings, fever, and abdominal pain, progressively worsening in the past year. She had already undergone several surgical drainages and six months of treatment with ciprofloxacin, with no improvement. Breast ultrasound and MRI were performed, which revealed a large fluid collection, with several small abscesses and surrounding adenopathies, complicated by multiple fistulae. The fluid was drained through needle aspiration, which was found to be sterile for bacteria, mycobacteria, and fungi. A lymph node biopsy showed necrosis without granulomas, and the biopsy culture was positive for *Mycobacterium tuberculosis *(Mt). This case shows how a rare manifestation can simulate bacterial breast abscesses or cancer, and hence a high index of suspicion is necessary to reach the correct diagnosis and ensure appropriate treatment delivery in these patients.

## Introduction

Breast tuberculosis (BTB) is an extremely rare presentation of a very common disease [1], especially in developing countries and immunosuppressed patients, such as those infected with HIV [2]. Migrants arriving in Portugal frequently originate from Portuguese-speaking African countries, such as Angola, due to historic reasons and existing health-related government protocols between these countries [3]. This leads to a higher incidence of severe or atypical presentations of these endemic infections, compared to the more frequent pulmonary and ganglionar tuberculosis (TB) seen in Portugal. The case presented here is an example of this phenomenon.

## Case presentation

**Figure 1:**
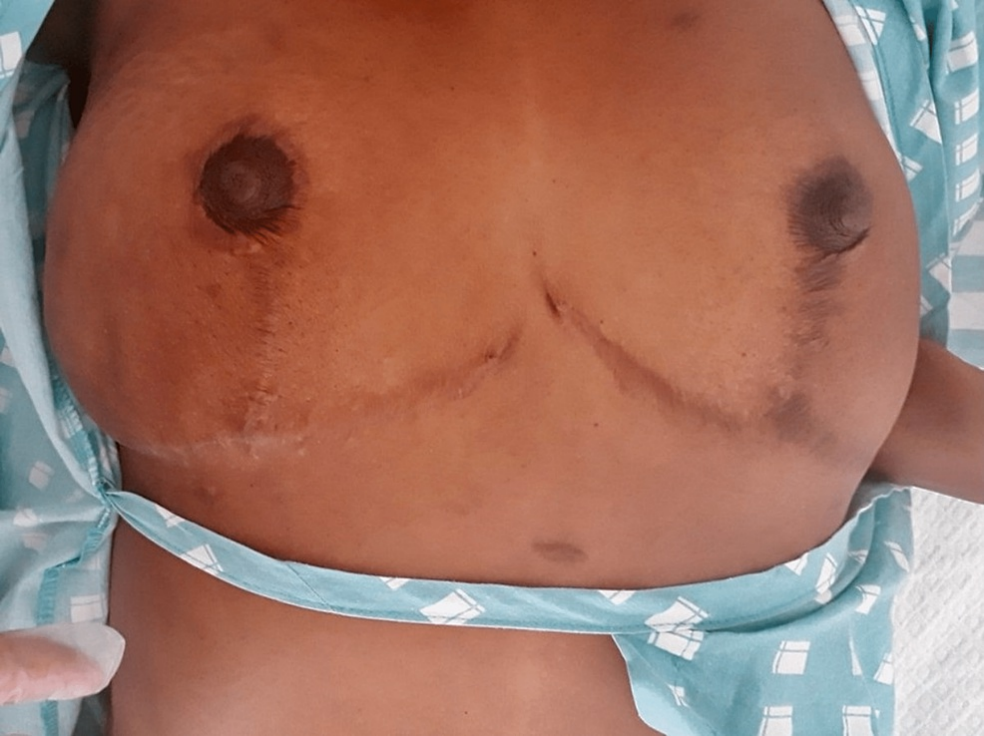
Breast asymmetry, with right breast enlargement in external quadrants

**Figure 2:**
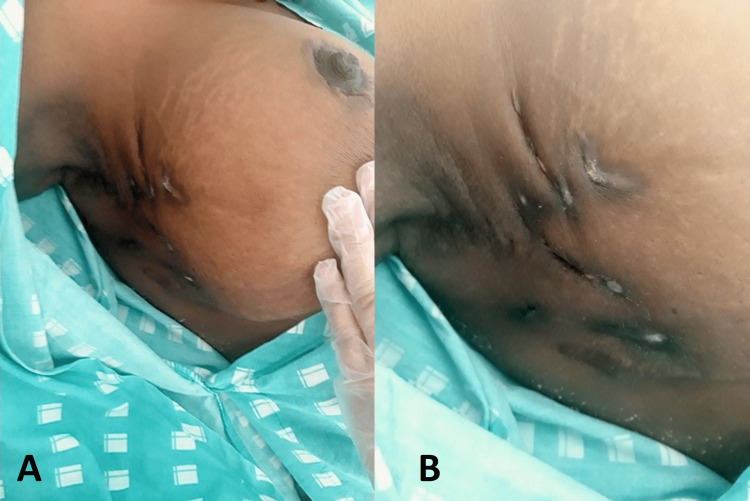
Multiple fistulae with purulent discharge on the external aspect of the right breast (A) The enlargement and (B) fistulae in detail

A 36-year-old Angolan woman, working in accounting, was referred to our hospital for a painful right breast mass associated with purulent discharge. Her symptoms had started one year prior, with the appearance of a warm, painful, swollen right axillary mass, and she had undergone surgical drainage in Angola. After one month, it had spread to the external right breast quadrants, associated with a fever of 39 ºC, and night sweats. Since then, she had undergone three additional surgical drainages of the mass and performed aerobic cultures only, once isolating *Klebsiella pneumoniae*, for which she had been prescribed six months of ciprofloxacin (unknown dosage). However, the swelling and pain had worsened and several fistulae had spontaneously appeared nearby (Figures 1, 2), from which a thick yellow exudate had started draining. She denied having any involuntary weight loss, lack of appetite, cough, or contralateral breast involvement. Her family history was unremarkable with regard to cancer.

**Figure 3:**
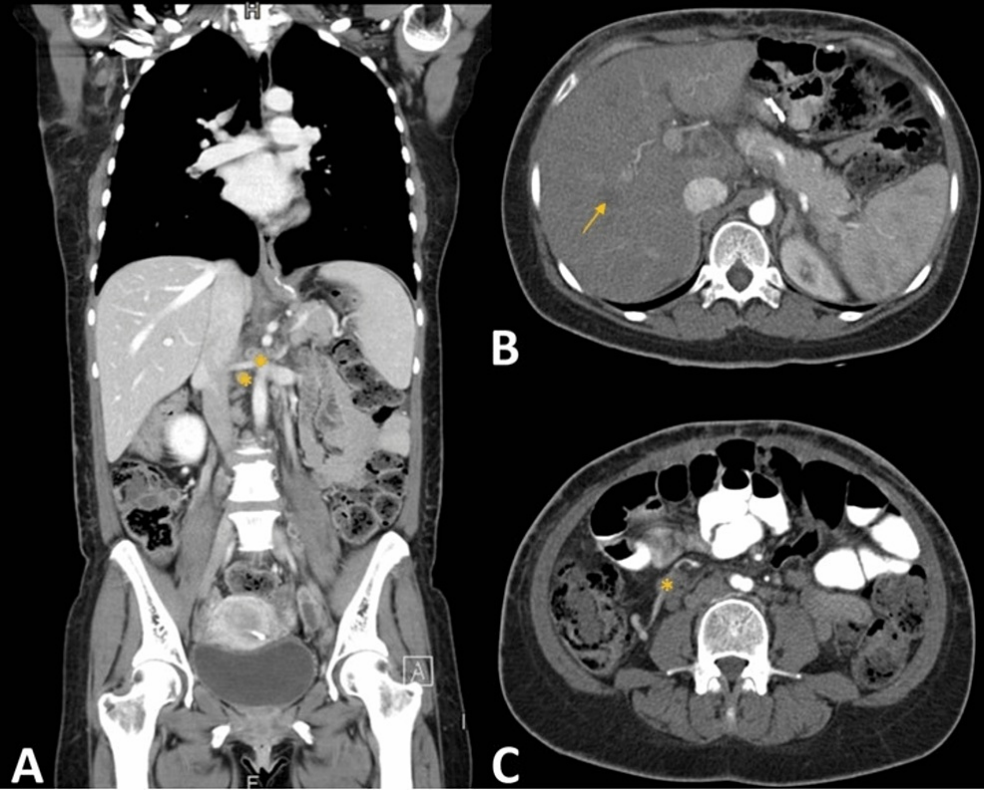
CT showing mediastinum, celiac, mesenteric, and latero-aortic adenomegalies, with necrotic center (*), and hepatosplenomegaly with hepatic nodular areas (arrow) (A) Coronal plane. (B) Transverse plane at the liver level. (C) Transverse plane at a lower level CT: computed tomography

On admission, she showed hypotension (blood pressure of 96/55 mmHg), sinus tachycardia (heart rate of 130 bpm), fever (38.9 ºC), pallor, and emaciation; she had keloid breast and abdominal scars and pain on deep palpation of her left upper abdominal quadrant but no peritoneal irritation. Her right breast was significantly enlarged, due to a dense adherent mass, with several fistula orifices spreading from the breast to the axilla, two of which with active purulent discharge. Her initial blood workup showed microcytic and hypochromic anemia (hemoglobin of 8.9 g/dL), neutrophilic leukocytosis (12,700/uL), elevated C-reactive protein (CRP) of 19.8 mg/dL, and erythrocyte sedimentation rate (ESR) of 119 mm/h. Her HIV test was negative. CT showed multiple mediastinal, celiac, mesenteric, and latero-aortic adenomegalies with a hypodense necrotic center, as well as hepatosplenomegaly, with ill-defined hypodense, hypocaptant nodular areas in the liver (Figure 3).

**Figure 4:**
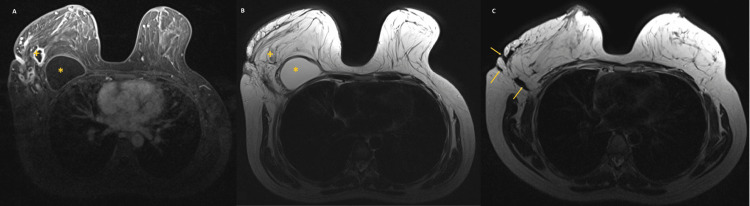
Breast MRI showing a collection (*) with thickened wall and gadolinium enhancement, several smaller abscesses (+), and fistulae (arrow) (A) T1-weighted. (B, C) T2-weighted MRI: magnetic resonance imaging

The mammography and breast ultrasound showed a homogeneous hypoechoic image of 90x37x69 mm, with a regular 2.8-mm septum; adjacent to it were irregular fistulae and adenomegalies, classified as BI-RADS 3 on the right. A breast MRI revealed a massive collection with thickened wall with gadolinium enhancement, and several smaller abscesses on a more superficial plane and fistulae, suggesting an extensive inflammatory process (Figure 4).

The patient was started on piperacillin-tazobactam, assuming bacterial abscess. Complete needle aspiration of the collection plus axillary core biopsies were performed, and *Mycobacterium tuberculosis* (Mt) was actively sought, due to the scrofulous appearance of the skin. The serohematic aspirate was negative for acid-fast bacteria in the Ziehl-Neelsen stain, as were culture and polymerase chain reaction (PCR) with cartridge-based nucleic acid amplification test for Mt. The biopsy showed fibroadipose tissue and necrotic material without granulomas. The biopsy’s culture was positive for Mt in both Löwenstein-Jensen medium and BACTEC^TM ^MGIT^TM^, showing no drug resistance in Middlebrook 7H10, thereby indicating a diagnosis of BTB. Lung TB was ruled out, with bronchial secretions and bronchoalveolar lavage being negative for Mt in Löwenstein-Jensen, BACTEC^TM ^MGIT^TM,^ and PCR. The patient's four-year-old son, whom she had breastfed for one year, had a negative TB screening.

The patient was started on daily weight-adjusted isoniazid (H) 300 mg, rifampin (R) 600 mg, pyrazinamide (Z) 1000 mg, and ethambutol (E) 800 mg, plus pyridoxine 40 mg. After consulting with the surgery team, we prioritized minimally invasive procedures, to reduce additional trauma to an already severe keloid scarring process, reserving surgery in case of refractoriness.

Her condition significantly improved in the first month of treatment with HRZE, with remission of fever and night sweats, ceasing of fistula drainage, and breast size reduction. Her anemia improved and ESR and CRP returned to normal levels (9 mm/h and 0.17 mg/dL, respectively). She completed 12 months of antituberculosis regimen (two months of HRZE followed by 10 months of HR), with complete resolution of symptoms, the disappearance of liver lesions, and the return of spleen size to normal; her lymph nodes decreased in size, despite maintaining a necrotic center.

## Discussion

TB is caused by Mt and is mostly transmitted through inhalation of particles produced by patients with a lung infection [4]. It was considered to be the leading infectious cause of death by the World Health Organization until the emergence of the coronavirus disease 2019 (COVID-19) pandemic [4]. While it mostly affects the lung, extrapulmonary involvement is also frequent, especially in HIV coinfection, representing 50% of cases [5]. Breast involvement in TB is extremely rare, estimated to account for only 0.025-0.1% of all TB cases [1]. Several risk factors have been identified, such as trauma, lactation, multiparity, and HIV [6], which can create an entry point to the breast tissue or augment duct permeability. Primary BTB can result from direct inoculation (trauma or ascending duct infection) [7], while secondary cases stem from lymphatic centripetal infection, spreading from contiguous structures (thoracic wall, pleura) or hematogenous seeding [8]. However, Nagashima (1925) examined 34 breast tissue samples of female patients who had died of TB (including the miliary form), and found no evidence of BTB, leading to the assumption that breast tissue is somewhat resistant to TB, even with bloodstream infection [9].

Our patient had two risk factors for direct inoculation: recent breast surgery and breastfeeding of her third child. However, the patient's symptoms had started with an axillary mass, suggesting TB lymphadenitis, which later spread to the ipsilateral breast. The patient also had other affected organs, but curiously not the lung, and hence we postulate that the lymphadenitis was the first site of infection, followed by systemic dissemination. Tewari and Shukla (2005) [7] proposed a disease classification comprising nodulocaseous tubercular mastitis (in which a slowly growing mass with possible fistulae develops), disseminated/confluent tubercular mastitis (associated with a large area of the breast being affected with small fluid collections, skin inflammation, and fistulae openings), and tubercular breast abscess (in which a single, large fluid collection forms). This patient appeared to have an overlap between the latter two, with multiple small fistulizing abscesses and a large fluid collection.

Females represent 98.6% of BTB cases, with a mean age at presentation of 29 years; unilateral upper breast quadrants were affected in 75% of cases [6]. An isolated lump is the most frequent presentation, and skin fistulae appear in one-quarter of cases, while constitutional symptoms are present in less than a third of patients [6]. In this case, the presence of a liquid collection would suggest bacterial mastitis, but the aspirate was sterile for aerobic/anaerobic bacteria, which, together with the failure of broad-spectrum antibiotics, should immediately raise suspicion for BTB. The fact that the aspirated fluid was negative for direct smear exam, Mt culture, and PCR, and that only the biopsy culture was positive in Lowenstein-Jensen and BACTEC medium (despite the absence of granulomas), makes it analogous to other cases of BTB [6]. Although the isolation of Mt is considered the gold standard for diagnosis, its sensitivity ranges from 25 to 58% [6], and some authors endorse diagnosing BTB based on the presence of caseous granulomatosis alone [10].

Our patient completed 12 months of antituberculous therapy because she still complained of breast pain and swelling and general malaise at the end of the initially scheduled six months of treatment. Surgery was not necessary in our case, which reinforces the perception that it is mostly an important diagnostic tool, especially when cancer is suspected, or when severe ulceration or abscedation is present [6]. When used as the sole treatment modality, surgery is bound to fail and aggravate progressive tissue destruction.

## Conclusions

BTB is a rare disease, frequently presenting as a slowly growing mass, which can be confused with bacterial mastitis or abscess in young patients and cancer in older patients. This often leads to delays in proper diagnosis and treatment, which makes the condition significantly different from these other entities. BTB’s diagnosis can be extremely challenging, with low-yield diagnostic tools, and is only possible if a high index of suspicion is maintained by clinicians, and the diagnosis can be even more difficult in countries with low TB incidence, such as Portugal.
